# Trends and Perspectives in Biosensing and Diagnosis

**DOI:** 10.3390/bios14100499

**Published:** 2024-10-12

**Authors:** Yan Zhang, Sai Bi, Qin Xu, Yingju Liu

**Affiliations:** 1 School of Chemistry and Chemical Engineering, University of Jinan, Jinan 250022, China; 2 College of Chemistry and Chemical Engineering, Key Laboratory of Shandong Provincial Universities for Functional Molecules and Materials, Qingdao University, Qingdao 266071, China; 3 Institute of Innovation Materials and Energy, School of Chemistry and Chemical Engineering, Yangzhou University, Yangzhou 225002, China; 4 College of Materials and Energy, South China Agricultural University, Guangzhou 510642, China

Biosensors are attractive tools for detecting molecules and small particles, as they can produce rapid, sensitive, and specific signals [1,2]. Recently, innovative research on nanomaterials, advanced manufacturing technologies, and interdisciplinary collaboration has significantly facilitated the development of simple, cost-effective diagnostic tools [3,4,5], allowing for immediate analysis and interpretation of complex samples. As a consequence, biosensing devices have been fabricated to provide rich information for applications in home healthcare [6,7,8], food quality control [9,10,11], environmental monitoring [12,13,14], and emergency security [15,16].

Generally, biosensors comprise three key components, including the target recognition element, transducer, and signal processing element. To obtain better output signals, various strategies have been introduced into the field of biosensing to improve the performance of different components [17,18]. For instance, nanozymes with intrinsic enzyme-like properties and better stability under a harsh environment have been widely employed as ideal recognition elements in the biosensing field, effectively overcoming the inactivation of natural enzymes [19,20,21]. In addition, metal–organic frameworks and their derivatives have been utilized as ideal carriers for the fabrication of biosensing interfaces by dispersing electrochemical or photochemical active substances [22,23]. Moreover, the ingenious and rational integration of signal amplification strategies can remarkably enhance the sensitivity of biosensing systems, allowing for rapid and accurate detection results [24,25]. As for the signal acquisition process, by integrating mobile devices into biosensing platforms [26,27], real-time monitoring of target conditions has been realized, which lays a good foundation for the point-of-care of diagnosis. It is foreseeable that the rapid disease detection based on biosensors can be realized soon, allowing individuals to take control of their own health while providing healthcare professionals with valuable data for decision-making [28,29].

Despite significant advances in biosensing and diagnosis, integrating biosensors into practice still requires robust validation studies to determine their effectiveness and reliability. It is essential to strengthen collaboration between academia, industry, and regulatory bodies to facilitate the translation of laboratory innovations into real-world applications [30,31,32]. Moreover, the convergence of machine learning, big data analytics, and the Internet of Things is poised to revolutionize the field of biosensing and diagnosis [33,34]. For example, machine learning algorithms can analyze the vast datasets generated by biosensors, identifying patterns and correlations that may not be obvious through traditional statistical approaches [35]. This capability enhances the accuracy of diagnostics, enabling personalized treatment plans tailored to the needs of individual patients. Following the evolving consumer demand and emerging technologies, it is believed that significant advances and major breakthroughs will predictably occur in this attractive field.

With the purpose of witnessing recent exciting achievements in such an attractive field, this Special Issue reports the innovations in a variety of detection techniques in this area, including electrochemistry [36], photoelectrochemistry [37], fluorescence [38], etc. It consists of 28 research articles, 3 communications, and 4 reviews, covering almost all the applications of biosensing and diagnosis mentioned above. It is believed that this Special Issue will be of great interest to early career researchers who are working in the domains of analytical chemistry, biotechnology, bioelectronics, and nanomedicine, and promote further high-quality research in such emerging fields.
